# Update: Influenza Activity in the United States During the 2018–19 Season and Composition of the 2019–20 Influenza Vaccine

**DOI:** 10.15585/mmwr.mm6824a3

**Published:** 2019-06-21

**Authors:** Xiyan Xu, Lenee Blanton, Anwar Isa Abd Elal, Noreen Alabi, John Barnes, Matthew Biggerstaff, Lynnette Brammer, Alicia P. Budd, Erin Burns, Charisse N. Cummings, Shikha Garg, Rebecca Kondor, Larisa Gubareva, Krista Kniss, Sankan Nyanseor, Alissa O’Halloran, Melissa Rolfes, Wendy Sessions, Vivien G. Dugan, Alicia M. Fry, David E. Wentworth, James Stevens, Daniel Jernigan

**Affiliations:** 1 Influenza Division, National Center for Immunization and Respiratory Diseases, CDC.

Influenza activity* in the United States during the 2018–19 season (September 30, 2018–May 18, 2019) was of moderate severity (*1*). Nationally, influenza-like illness (ILI)^†^ activity began increasing in November, peaked during mid-February, and returned to below baseline in mid-April; the season lasted 21 weeks,^§^ making it the longest season in 10 years. Illness attributed to influenza A viruses predominated, with very little influenza B activity. Two waves of influenza A were notable during this extended season: influenza A(H1N1)pdm09 viruses from October 2018 to mid-February 2019 and influenza A(H3N2) viruses from February through May 2019. Compared with the 2017–18 influenza season, rates of hospitalization this season were lower for adults, but were similar for children. Although influenza activity is currently below surveillance baselines, testing for seasonal influenza viruses and monitoring for novel influenza A virus infections should continue year-round. Receiving a seasonal influenza vaccine each year remains the best way to protect against seasonal influenza and its potentially severe consequences.

## Virus Surveillance

U.S. World Health Organization (WHO) collaborating laboratories and National Respiratory and Enteric Virus Surveillance System laboratories, which include both clinical and public health laboratories throughout the United States, contribute to virologic surveillance for influenza. During September 30, 2018–May 18, 2019, clinical laboratories tested 1,145,555 specimens for influenza virus; among these, 177,039 (15.5%) tested positive, including 167,529 (95.0%) for influenza A and 9,510 (5.0%) for influenza B. The percentage of specimens testing positive for influenza each week ranged from 1.7% to 26.2%.

Nationally, the percentage of clinical laboratory–tested specimens positive for influenza virus peaked during the weeks ending February 9–March 16 (surveillance weeks 6–11) (range = 25.1%–26.2%). Regionally,^¶^ the week of peak clinical laboratory influenza positivity varied, ranging from the week ending December 15, 2018 (week 50) to the week ending March 16, 2019 (week 11).

**FIGURE 1:**
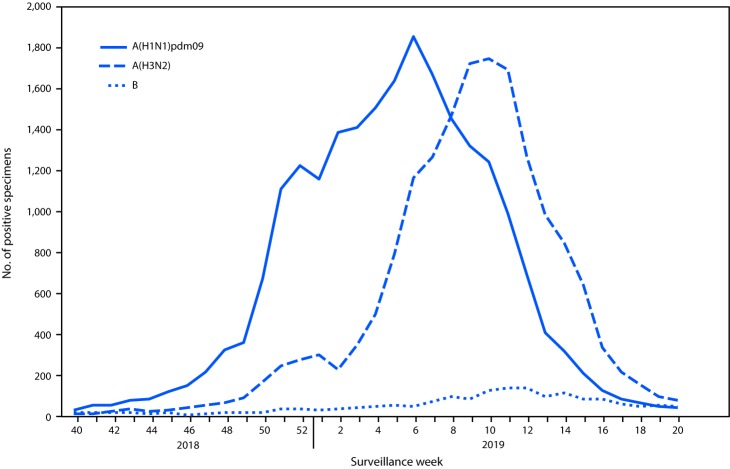
Number* of respiratory specimens testing positive for influenza reported to CDC by public health laboratories, by influenza virus type, subtype,^†^ and surveillance week – United States, September 30, 2018–May 18, 2019^§^ * N = 40,674. ^†^ 1,629 influenza A viruses not subtyped are excluded. ^§^ As of June 14, 2019.

Public health laboratories tested 80,993 specimens during September 30, 2018–May 18, 2019; among these specimens, 42,303 (52.2%) were positive for influenza viruses, including 40,624 (96.0%) that were positive for influenza A and 1,679 (4.0%) for influenza B. Among the 38,995 seasonal influenza A viruses subtyped, 22,084 (56.6%) were influenza A(H1N1)pdm09, and 16,991 (43.6%) were influenza A(H3N2). Influenza B lineage information was available for 1,105 (65.8%) influenza B viruses; 406 (36.7%) of those were B/Yamagata lineage, and 699 (63.3%) were B/Victoria lineage. Whereas influenza A(H1N1)pdm09 viruses accounted for the majority of circulating viruses nationwide from October 2018 to mid-February 2019, influenza A(H3N2) viruses were detected more frequently than were A(H1N1)pdm09 viruses beginning in late February nationally (Figure 1) and in all 10 U.S. Health and Human Services (HHS) regions by the end of March 2019. For the season overall, influenza A(H3N2) viruses predominated in HHS Regions 4, 6, and 7, and influenza A(H1N1)pdm09 viruses predominated in the remaining seven regions.

Among 38,564 (91.2%) patients whose test results were positive for seasonal influenza virus by public health laboratories and for whom age data were available, 4,844 (12.6%) were aged 0–4 years; 12,508 (32.4%) were aged 5–24 years; 13,382 (34.7%) were aged 25–64 years; and 7,830 (20.3%) were aged ≥65 years. Influenza A(H1N1)pdm09 virus was the most frequently reported virus among persons aged 0–4 years (57.1%) and 25–64 years (63.2%), whereas influenza A(H3N2) virus was the most commonly reported virus among persons aged 5–24 years (48.8%) and ≥65 years (51.3%). The age group with the largest proportion of reported influenza B viruses (6.3%) was persons aged 5–24 years.

## Antigenic and Genetic Characterization of Influenza Viruses

Genetic characterization was carried out using next-generation sequencing, and the genomic data were analyzed and submitted to public databases (GenBank: https://www.ncbi.nlm.nih.gov/genbank or EpiFlu: https://www.gisaid.org/). Antigenic characterizations were carried out by hemagglutination inhibition assays or virus neutralization–based focus reduction assays to evaluate whether genetic changes in circulating viruses affected antigenicity; substantial differences could affect vaccine effectiveness. CDC genetically characterized 2,750 influenza viruses collected and submitted** by U.S. laboratories since September 30, 2018, including 1,251 influenza A(H1N1)pdm09 viruses, 1,024 influenza A(H3N2) viruses, and 475 influenza B viruses. A subset of these viruses also was antigenically characterized. Phylogenetic analysis of the hemagglutinin (HA) gene segments from the 1,251 characterized A(H1N1)pdm09 viruses determined that all belonged to genetic subclade 6B.1A, which evolved from clade 6B.1. Among 331 antigenically characterized A(H1N1)pdm09 viruses, 318 (96.1%) were well inhibited (reacting at titers that were within fourfold of the homologous virus titer) by ferret antisera raised against A/Michigan/45/2015 (6B.1), the cell culture–propagated reference virus representing the A(H1N1)pdm09 component for the 2018–19 Northern Hemisphere influenza vaccines.

Phylogenetic analysis of the HA gene segments of 1,204 sequenced influenza A(H3N2) viruses indicated cocirculation of multiple clades/subclades. Circulating viruses possessed HA gene segments that belonged to clade 3C.2a (66; 6.4%), subclade 3C.2a1 (201; 19.6%), or clade 3C.3a (757; 73.9%). The frequency of 3C.3a viruses increased from 12.7% of the A(H3N2) viruses collected and sequenced by November 2018 to 81.9% of those collected and sequenced during December 2018–May 2019. Among the 505 A(H3N2) viruses antigenically characterized by focus reduction assays with ferret antisera, 191 (37.8%) were well inhibited by ferret antisera raised against A/Singapore/INFIMH-16-0019/2016 (3C.2a1), a cell culture–propagated reference virus representing the A(H3N2) component of 2018–19 Northern Hemisphere influenza vaccines. However, only 43 (11%) of the 388 viruses tested were well inhibited by antiserum raised against egg-propagated A/Singapore/INFIMH-16-0019/2016 reference virus, likely because of egg-adaptive amino acid changes in the HA protein of the egg-propagated virus. Three hundred fourteen (62.2%) viruses were poorly inhibited by ferret antiserum raised against cell culture–propagated A/Singapore/INFIMH-16-0019/2016 reference virus (at titers that were reduced eightfold or more when compared with the homologous virus); among those viruses, 312 (99.4%) belonged to clade 3C.3a, the prevalence of which increased throughout the season.

Phylogenetic analysis of 203 influenza B/Yamagata lineage viruses determined that the HA gene segments belonged to clade Y3. All 178 B/Yamagata lineage viruses antigenically characterized were well inhibited by ferret antiserum raised against cell culture–propagated B/Phuket/3073/2013, the reference virus representing the B/Yamagata lineage component of quadrivalent vaccines for the 2018–19 Northern Hemisphere influenza season.

Multiple genetically and antigenically distinct B/Victoria lineage viruses cocirculated during the 2018–19 season. Viruses with a two-amino acid deletion (162–163) in the HA protein belong to subclade V1A.1, and viruses with a three-amino acid deletion (162–164) in the HA protein belong to subclade V1A-3Del. Among the 272 influenza B/Victoria lineage viruses sequenced and phylogenetically analyzed, the HA gene segment belonged to genetic clade V1A (40; 14.7%), subclade V1A.1 (137; 50.4%), or subclade V1A-3Del (95; 34.9%). Among 191 B/Victoria lineage viruses antigenically characterized, 147 (79.1%) were well inhibited by ferret antiserum raised against cell culture–propagated B/Colorado/06/2017-like V1A.1 reference virus representing the B/Victoria lineage component of the vaccines for the 2018–19 Northern Hemisphere influenza season. Among the 44 (20.9%) viruses that reacted poorly, 17 were antigenically related to the previous vaccine virus B/Brisbane/60/2008 and belonged to clade V1A, and 27 belonged to subclade V1A-3Del.

## Antiviral Susceptibility of Influenza Viruses

Testing of seasonal influenza A(H1N1)pdm09, influenza A(H3N2), and influenza B viruses for resistance to the neuraminidase inhibitors oseltamivir, zanamivir, and peramivir is performed at CDC using next-generation sequencing analysis, a functional assay (*2*), or both. Neuraminidase sequences of viruses are examined for the presence of amino acid substitutions previously associated with reduced or highly reduced inhibition by any of the three neuraminidase inhibitors.^††^ The amino acid substitution H275Y in A(H1N1)pdm09 viruses is considered clinically relevant because of the frequency of occurrence and the availability of clinical data demonstrating a reduced treatment efficacy; however, other amino acid substitutions have been observed less frequently and caused reduced susceptibility in vitro, but with less clear clinical significance (*2*).

A total of 2,699 influenza virus specimens, including 1,240 influenza A(H1N1)pdm09, 1,016 influenza A(H3N2), 252 influenza B/Victoria, and 191 influenza B/Yamagata viruses collected in the United States since October 1, 2018, were tested for resistance to oseltamivir, zanamivir, and peramivir. Five (0.3%) influenza A(H1N1)pdm09 viruses had the amino acid substitution H275Y and displayed highly reduced inhibition by oseltamivir and peramivir. In addition, four (0.3%) influenza A(H1N1)pdm09 viruses displayed some reduction in inhibition by oseltamivir, and two influenza B viruses (0.4%) from different lineages had the amino acid substitution H273Y and displayed highly reduced inhibition by peramivir.

During the 2018–19 influenza season, CDC began to test seasonal influenza viruses for resistance to the PA cap-dependent endonuclease inhibitor baloxavir using next-generation sequencing analysis, a phenotypic assay (*3*), or both. PA protein sequences were examined for the presence of amino acid substitutions previously associated with decreased susceptibility or resistance to baloxavir (*3*).

Among 2,673 influenza virus specimens, including 1,213 influenza A(H1N1)pdm09, 1,007 influenza A(H3N2), 255 influenza B/Victoria, and 198 influenza B/Yamagata viruses collected in the United States since October 1, 2018, and tested genetically for resistance to baloxavir, none contained amino acid substitutions in the PA protein previously associated with decreased susceptibility to baloxavir. All 191 influenza viruses tested by a phenotypic assay were susceptible to baloxavir.

## Composition of the 2019–20 Influenza Vaccines

Vaccine recommendations were made based on factors including data from global influenza virologic and epidemiologic surveillance, genetic characterization, antigenic characterization, and the candidate vaccine viruses that are available for production. WHO recommended the Northern Hemisphere 2019–20 influenza vaccine composition (*4*), and the Food and Drug Administration’s Vaccines and Related Biologic Products Advisory Committee subsequently made the influenza vaccine composition recommendation for the United States (*5*,*6*). Both agencies recommend that influenza trivalent vaccines contain an A/Brisbane/02/2018 A(H1N1)pdm09-like virus, an A/Kansas/14/2017 A(H3N2)-like virus, and a B/Colorado/06/2017-like (B/Victoria lineage) virus. The quadrivalent vaccine recommendation included the trivalent vaccine viruses and a B/Phuket/3073/2013-like (B/Yamagata lineage) virus. The A(H1N1)pdm09 and A(H3N2) recommendations are an update to the 2018–19 Northern Hemisphere vaccines. The decision to update the A(H1N1)pdm09 component was made because of genetic and antigenic characterization data using individual postvaccination human sera, which demonstrated significantly reduced titers (eightfold or greater) to recent 6B.1A viruses, compared with the titers against the A/Michigan/45/2015 vaccine virus (*5*). The decision to update the A(H3N2) component was made to address antigenic drift of the virus with emergence and spread of A/Kansas/14/2017-like viruses (3C.3a) (*6*).

## Outpatient Illness Surveillance

**FIGURE 2:**
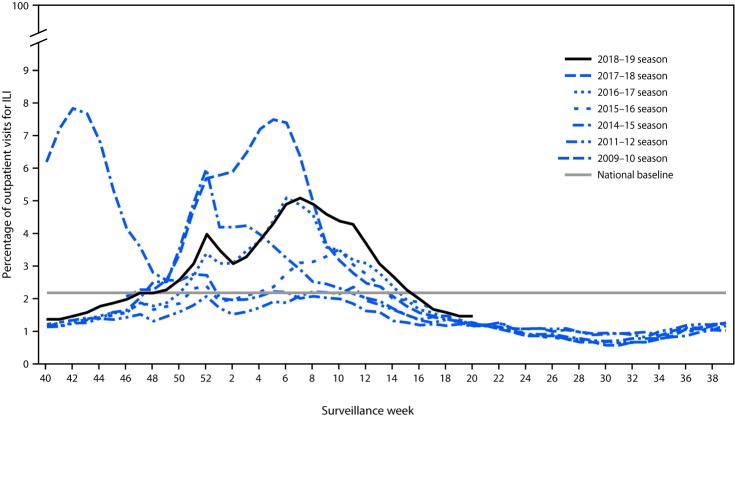
Percentage of outpatient visits for influenza-like illness (ILI)* reported to CDC, by surveillance week — U.S. Outpatient Influenza-like Illness Surveillance Network, 2018–19^†^ influenza season and selected previous influenza seasons * Defined as fever (temperature of ≥100°F [≥37.8°C], oral or equivalent) and cough or sore throat, without a known cause other than influenza. ^†^ As of June 14, 2019.

Nationally, the weekly percentage of outpatient visits for ILI to health care providers participating in the U.S. Outpatient Influenza-like Illness Surveillance Network (ILINet) was at or above the national baseline^§§^ level of 2.2% for 21 consecutive weeks (weeks 47–15) during the 2018–19 season (Figure 2). The percentage of outpatient ILI visits peaked at 5.1% during the week ending February 16, 2018 (week 7).

ILINet data are used to produce a weekly jurisdiction-level measure of ILI activity,^¶¶^ ranging from minimal to high. The number of jurisdictions reporting high ILI activity peaked during the week ending February 23, 2019 (week 8) when 33 (61%) of 54 jurisdictions (50 states, New York City, the District of Columbia, Puerto Rico, and U.S. Virgin Islands) experienced high ILI activity.

## Geographic Spread of Influenza Activity

State and territorial epidemiologists report the geographic distribution of influenza in their jurisdictions through a weekly influenza activity code.*** During the 2018–19 season, the peak number of jurisdictions reporting widespread activity in a single week was 50 (93%); this occurred during week 8 (week ending February 23, 2019).

## Influenza-Associated Hospitalizations

CDC monitors hospitalizations associated with laboratory-confirmed influenza infections through the Influenza Hospitalization Surveillance Network (FluSurv-NET),^†††^ which covers approximately 27 million persons (9% of the U.S. population). During October 1, 2018–April 30, 2019, a total of 18,847 laboratory-confirmed influenza-related hospitalizations were reported (cumulative incidence for all age groups = 65.3 per 100,000 population). The overall peak occurred during the week ending March 16, 2019 (week 11). The hospitalization rate was highest among persons aged ≥65 years, who accounted for approximately 47% of reported influenza-associated hospitalizations. By age group, the cumulative hospitalization rate per 100,000 population was 72.0 among children aged 0–4 years, 20.4 among children and adolescents aged 5–17 years, 25.8 among adults aged 18–49 years, 80.7 among adults aged 50–64 years, and 221.7 among adults aged ≥65 years. Among all influenza-associated hospitalizations, 17,993 (95.5%) were associated with influenza A virus, 727 (3.9%) with influenza B virus, 41 (0.2%) with influenza A virus and influenza B virus coinfection, and 86 (0.5%) with influenza virus for which the type was not determined. Among 6,360 (35.3%) with influenza A subtype information, 3,367 (52.9%) were influenza A(H1N1)pdm09 viruses, and 2,993 (47.1%) were influenza A(H3N2) viruses.

Complete medical chart abstraction data in FluSurv-NET will not be finalized until later in 2019; however, as of June 13, 2019, data were available for 7,531 (40.0%) hospitalized adults and children with laboratory-confirmed influenza. Among 6,399 hospitalized adults with information on underlying medical conditions, 92.6% had at least one reported underlying medical condition that placed them at high risk^§§§^ for influenza-associated complications. The most commonly reported underlying medical conditions among adults were cardiovascular disease (45.0%), metabolic disorders (42.9%), obesity (39.4%), and chronic lung disease (29.9%). Among 1,132 hospitalized children with such information, 55.0% had at least one underlying medical condition; those most commonly reported were asthma (27.1%) and neurologic disorder (14.7%). Among 759 hospitalized females aged 15–44 years with information on pregnancy status, 152 (28.7%) were pregnant.

## Pneumonia and Influenza-Associated Mortality

CDC tracks pneumonia and influenza (P&I)–attributed deaths through CDC’s National Center for Health Statistics (NCHS) Mortality Surveillance System data. The percentages of deaths attributed to P&I are released 2 weeks after the week of death to allow for collection of sufficient data to produce a stable P&I mortality percentage. During the 2018–19 season, according to NCHS data, the proportion of deaths attributed to P&I was at or above the epidemic threshold^¶¶¶^ for 10 weeks during the weeks ending January 5–26, 2019 (weeks 1–4), the weeks ending February 16–March 2, 2019 (weeks 7–9), and the weeks ending March 16–30, 2019 (weeks 11–13). Nationally, mortality attributed to P&I peaked two times at 7.7% during the weeks ending February 23 (week 8) and March 16, 2019 (week 11).

## Influenza-Associated Pediatric Mortality

During September 30, 2018–May 18, 2019, 116 laboratory-confirmed influenza-associated pediatric deaths were reported to CDC from Chicago, New York City, and 41 states. Two deaths occurred in non-U.S. residents. Twenty-five (22%) of the deaths were associated with influenza A(H3N2) infection, 43 (37%) with influenza A(H1N1)pdm09, 39 (34%) with an influenza A virus for which no subtyping was performed, eight (7%) with an influenza B virus, and one (1%) with an influenza virus for which the type was not determined. The mean age of the pediatric deaths reported this season was 6.1 years (range = 2 months–17 years); 75 (66%) children died after admission to the hospital. Among the 104 children with a known medical history, 53 (51%) had at least one underlying medical condition recognized by the Advisory Committee on Immunization Practices (ACIP) as placing them at high risk for influenza-related complications. Among the 89 children who were eligible for influenza vaccination (age ≥6 months at date of onset) and for whom vaccination status was known, 30 (34%) had received at least 1 dose of influenza vaccine before illness onset (25 were fully vaccinated according to 2018 ACIP recommendations, and five had received 1 of 2 recommended doses).

## Severity Assessment

In 2017, CDC implemented a new methodology to classify influenza season severity using three indicators: 1) the percentage of visits to outpatient clinics for ILI (from ILINet); 2) the rates of influenza-associated hospitalizations (from FluSurv-Net); and 3) the percentage of deaths resulting from pneumonia or influenza (from NCHS) (*1*). This approach uses data from past influenza seasons to calculate three intensity thresholds (https://www.cdc.gov/flu/professionals/classifies-flu-severity.htm). These intensity thresholds represent the historic chance that surveillance system data exceeded a certain threshold. CDC then classifies the severity of the current influenza season by determining which intensity threshold was exceeded by at least two of the peak values from these indicators. The severity of the 2018–19 season was thus classified as moderate overall, as well as by age group (for children and adolescents, adults, and older adults).

## Preliminary Estimates of Influenza Burden

CDC uses the cumulative rates of influenza-associated hospitalizations reported through FluSurv-NET and a mathematical model**** to estimate the number of persons who have been symptomatically ill with influenza who had a medical visit, were hospitalized, or died related to influenza. Using data available from October 1, 2018, to May 4, 2019, CDC estimates that influenza virus infection has caused 37.4 million–42.9 million symptomatic illnesses; 17.3 million–20.1 million medical visits; 531,000–647,000 hospitalizations; and 36,400–61,200 deaths in the United States.

## Discussion

The 2018–19 U.S. influenza season differed from recent seasons in that there were two waves of influenza A activity of similar magnitude during the season. Influenza A(H1N1)pdm09 viruses predominated overall and represented the most frequently detected influenza A virus from October 2018 to mid-February 2019; influenza A(H3N2) viruses were reported more frequently than were A(H1N1)pdm09 viruses from late February through mid-May 2019. The predominant influenza A virus also differed by geographic region and age group. In contrast to the number of influenza A viruses reported, the number of influenza B viruses reported was low, compared with previous seasons, accounting for 4% of influenza viruses reported by public health laboratories.

The 2018–19 influenza season was longer than recent influenza seasons, and ILI activity was at or above baseline for 21 consecutive weeks. Compared with hospitalization rates during the previous five influenza seasons, the 2018–19 cumulative influenza-associated hospitalization rate (65.3 per 100,000 population) was most similar to rates observed during 2014–15 (64.1) and 2016–17 (62.0) and well below those observed during 2017–18 (102.9). Hospitalization rates for children aged <17 years exceeded those during the 2013–14 through 2016–17 seasons and were similar to those during the 2017–18 season, whereas hospitalization rates for adults aged 18–64 years exceeded those in 2013–14 through 2016–17 but were less than those during the 2017–18 season. For persons aged ≥65 years, this season’s hospitalization rates were below those observed during the three most recent H3N2-predominant seasons (2014–15, 2016–17, and 2017–18) but higher than the two H1N1-predominant seasons (2013–14 and 2015–16). Compared with P&I-attributed mortality during the previous five seasons, 2018–19 P&I-attributed mortality was most similar to the 2015–16 season and was lower than that during the other four seasons.

Most of the influenza A(H1N1)pdm09 viruses characterized (using hemagglutination inhibition tests with ferret antisera) were antigenically similar to the cell culture–propagated reference virus representing the 2018–19 Northern Hemisphere influenza vaccine virus, but considerable genetic diversity among currently circulating influenza A(H1N1)pdm09 viruses belonging to clade 6B.1A was observed. The increased circulation of clade 3C.3a viruses strongly contributed to the increasing proportion of A(H3N2) viruses that were antigenically distinct from the reference virus representing the A(H3N2) vaccine component of the 2018–19 Northern Hemisphere vaccines. Viruses from clade 3C.3a were well inhibited by ferret antisera raised against recent 3C.3a cell culture–propagated reference viruses, including A/Kansas/14/2017, the reference virus representing the A(H3N2) component for the 2019–20 Northern Hemisphere influenza vaccines (*4*). All B/Yamagata lineage viruses and the majority of B/Victoria lineage viruses tested were antigenically similar to the reference viruses representing the components of vaccines for the 2018–19 Northern Hemisphere influenza season. However, B/Victoria lineage subclade V1A-3Del viruses, which were antigenically distinct from the B/Victoria lineage vaccine virus, were more frequently reported in the United States toward the end of the season. The majority (>99%) of influenza viruses collected and tested since October 1, 2018, were susceptible to oseltamivir and peramivir, and all tested viruses were susceptible to zanamivir and baloxavir.

Since the 2010–11 season, CDC estimates that during each influenza season, influenza virus infection has caused 9.3 million–49 million symptomatic illnesses, 4.3 million–23 million medical visits, 140,000–960,000 hospitalizations, and 12,000–79,000 deaths.^††††^ Preliminary estimates for the 2018–19 season fall within these ranges.

Receiving a seasonal influenza vaccine each year remains the best way to protect against seasonal influenza and its potentially severe consequences. Although seasonal influenza activity is currently below baseline, influenza illnesses are often reported during the summer. Influenza should be suspected in ill travelers returning from countries with ongoing influenza activity. Variant influenza infections associated with exposure to swine during animal exhibitions are reported each summer (*7*). Suspected variant influenza infections should be referred to state public health departments for testing. Treatment as soon as possible with influenza antiviral medications is recommended for patients with confirmed or suspected influenza who have severe, complicated, or progressive illness; who require hospitalization; or who are at high risk for influenza-associated complications (*8*). Providers should not rely on less sensitive assays such as rapid antigen detection influenza diagnostic tests to inform treatment decisions. Four influenza antiviral drugs are approved by the Food and Drug Administration for treatment of acute uncomplicated influenza within 2 days of illness onset and are recommended for use in the United States during the 2018–19 season: oseltamivir, zanamivir, peramivir, and baloxavir.

Influenza surveillance reports for the United States are posted online weekly (https://www.cdc.gov/flu/weekly). Additional information regarding influenza viruses, influenza surveillance, influenza vaccine, influenza antiviral medications, and novel influenza A infections in humans is available online (https://www.cdc.gov/flu).

**Summary**

**What is already known about this topic?**

CDC collects, compiles, and analyzes data on influenza activity and viruses in the United States.

**What is added by this report?**

The 2018–19 influenza season was a moderate severity season with two waves of influenza A activity of similar magnitude during the season: A(H1N1)pdm09 predominated from October 2018 to mid-February 2019, and A(H3N2) activity increased from mid-February through mid-May.

**What are the implications for public health practice?**

Receiving a seasonal influenza vaccine each year remains the best way to protect against seasonal influenza and its potentially severe consequences. Testing for seasonal influenza viruses and monitoring for emergence of antigenic drift variant viruses should continue year-round.

## Footnotes

* The CDC influenza surveillance system collects five categories of information from eight data sources: 1) virus surveillance (U.S. World Health Organization collaborating laboratories, the National Respiratory and Enteric Virus Surveillance System, and novel influenza A virus case reporting); 2) outpatient illness surveillance (U.S. Outpatient Influenza-like Illness Surveillance Network); 3) mortality (the National Center for Health Statistics Mortality Surveillance System and influenza-associated pediatric mortality reports); 4) hospitalizations (FluSurv-NET, which includes the Emerging Infections Program and surveillance in three additional states); and 5) summary of the geographic spread of influenza (state and territorial epidemiologist reports). https://www.cdc.gov/flu/weekly/fluactivitysurv.htm.

† Defined as a fever (temperature ≥100°F [≥37.8°C], oral or equivalent) and cough or sore throat, without a known cause other than influenza.

§ Data as of June 14, 2019.

¶ The 10 regions include the following jurisdictions: *Region 1:* Connecticut, Maine, Massachusetts, New Hampshire, Rhode Island, and Vermont; *Re*gi*on 2:* New Jersey, New York, Puerto Rico, and U.S. Virgin Islands; *Region 3:* Delaware, District of Columbia, Maryland, Pennsylvania, Virginia, and West Virginia; *Region 4:* Alabama, Florida, Georgia, Kentucky, Mississippi, North Carolina, South Carolina, and Tennessee; *Region 5:* Illinois, Indiana, Michigan, Minnesota, Ohio, and Wisconsin; *Region 6:* Arkansas, Louisiana, New Mexico, Oklahoma, and Texas; *Region 7:* Iowa, Kansas, Missouri, and Nebraska; *Region 8:* Colorado, Montana, North Dakota, South Dakota, Utah, and Wyoming; *Region 9:* Arizona, California, Hawaii, Nevada, American Samoa, Commonwealth of the Northern Mariana Islands, Federated States of Micronesia, Guam, Marshall Islands, and Palau; *Region 10:* Alaska, Idaho, Oregon, and Washington.

** Association of Public Health Laboratories. Influenza Virologic Surveillance Right Size Roadmap. https://www.aphl.org/AboutAPHL/publications/Documents/ID_July2013_Influenza-Virologic-Surveillance-Right-Size-Roadmap.pdf.

†† https://www.who.int/influenza/gisrs_laboratory/antiviral_susceptibility/NAI_Reduced_Susceptibility_Marker_Table_WHO.pdf?ua.

§§ The national and regional baselines are the mean percentages of visits for ILI during noninfluenza weeks for the previous three seasons plus two standard deviations. Noninfluenza weeks are defined as periods of ≥2 consecutive weeks during which each week accounted for <2% of the season’s total number of specimens that tested positive for influenza. National and regional percentages of patient visits for ILI are weighted according to state population. Use of the national baseline for regional data is not recommended.

¶¶ Activity levels are derived from the percentage of outpatient visits in a jurisdiction attributed to ILI and are compared with the average percentage of ILI visits that occur during weeks with little or no influenza virus circulation. Activity levels range from minimal, corresponding to ILI activity from outpatient clinics at or below the average, to high, corresponding to ILI activity from outpatient clinics much higher than the average. Because the clinical definition of ILI is nonspecific, not all ILI is caused by influenza; however, when combined with laboratory data, the information on ILI activity provides a clearer picture of influenza activity in the United States.

*** Levels of activity are 1) no activity; 2) sporadic: isolated laboratory-confirmed influenza cases or a laboratory-confirmed outbreak in one institution, with no increase in activity; 3) local: increased ILI, or two or more institutional outbreaks (ILI or laboratory-confirmed influenza) in one region of the state, with recent laboratory evidence of influenza in that region; virus activity no greater than sporadic in other regions; 4) regional: increased ILI activity or institutional outbreaks (ILI or laboratory-confirmed influenza) in two or more outbreaks, but fewer than half of the regions in the state with recent laboratory evidence of influenza in those regions; and 5) widespread: increased ILI activity or institutional outbreaks (ILI or laboratory-confirmed influenza) in at least half of the regions in the state, with recent laboratory evidence of influenza in the state.

††† FluSurv-NET conducts population-based surveillance for laboratory-confirmed, influenza-associated hospitalizations in children and adolescents aged <18 years (since the 2003–04 influenza season) and adults aged ≥18 years (since the 2005–06 influenza season). FluSurv-NET covers approximately 70 counties in the 10 Emerging Infections Program states (California, Colorado, Connecticut, Georgia, Maryland, Minnesota, New Mexico, New York, Oregon, and Tennessee) and additional Influenza Hospitalization Surveillance Project (IHSP) states. IHSP began during the 2009–10 season to enhance surveillance during the 2009 H1N1 pandemic. IHSP sites included Idaho, Iowa, Michigan, Oklahoma, and South Dakota during the 2009–10 season; Idaho, Michigan, Ohio, Oklahoma, Rhode Island, and Utah during the 2010–11 season; Michigan, Ohio, Rhode Island, and Utah during the 2011–12 season; Iowa, Michigan, Ohio, Rhode Island, and Utah during the 2012–13 season; and Michigan, Ohio, and Utah during the 2013–14, 2014–15, 2015–16, and 2016–17 seasons. Cumulative unadjusted incidence rates are calculated using CDC’s National Center for Health Statistics population estimates for the counties included in the surveillance catchment area. Laboratory confirmation is dependent on clinician-ordered influenza testing, and testing for influenza often is underused because of the poor reliability of rapid test results and greater reliance on clinical diagnosis for influenza. Therefore, cases identified as part of influenza hospitalization surveillance likely are an underestimation of the actual number of persons hospitalized with influenza.

§§§ Persons at higher risk include 1) children aged <2 years; 2) adults aged ≥65 years; 3) persons with chronic pulmonary conditions (including asthma), cardiovascular disease (except hypertension alone), renal, hepatic, hematologic (including sickle cell) disease, metabolic disorders (including diabetes mellitus), or neurologic and neurodevelopmental conditions (including disorders of the brain, spinal cord, peripheral nerves, and muscles, such as cerebral palsy, epilepsy [seizure disorders], stroke, intellectual disability [mental retardation], moderate to severe developmental delay, muscular dystrophy, or spinal cord injury); 4) persons with immunosuppression, including that caused by medications or by human immunodeficiency virus infection; 5) women who are pregnant or postpartum (within 2 weeks after delivery); 6) persons aged ≤18 years who are receiving long-term aspirin therapy; 7) American Indians/Alaska Natives; 8) persons with extreme obesity (i.e., body mass index ≥40); and 9) residents of nursing homes and other chronic care facilities.

¶¶¶ The seasonal baseline proportion of P&I deaths is projected using a robust regression procedure, in which a periodic regression model is applied to the observed percentage of deaths from P&I that were reported by the National Center for Health Statistics Mortality Surveillance System during the preceding 5 years. The epidemic threshold is set at 1.645 standard deviations above the seasonal baseline.

**** https://www.cdc.gov/flu/about/burden/preliminary-in-season-estimates.htm.

†††† https://www.cdc.gov/flu/about/burden/past-seasons.html.
